# BET family protein degraders poised to join the senolytic arsenal

**DOI:** 10.1038/s41392-020-0202-2

**Published:** 2020-06-10

**Authors:** Zufeng Guo, Hanjing Peng, Yongmei Xie

**Affiliations:** 1 Department of Pharmacology and Molecular Sciences, Johns Hopkins School of Medicine, Baltimore, MD USA; 2 The SJ Yan and HJ Mao Laboratory of Chemical Biology, Johns Hopkins School of Medicine, Baltimore, MD USA

**Keywords:** Chemical biology, Drug discovery, Molecular medicine

**Fig. 1:**
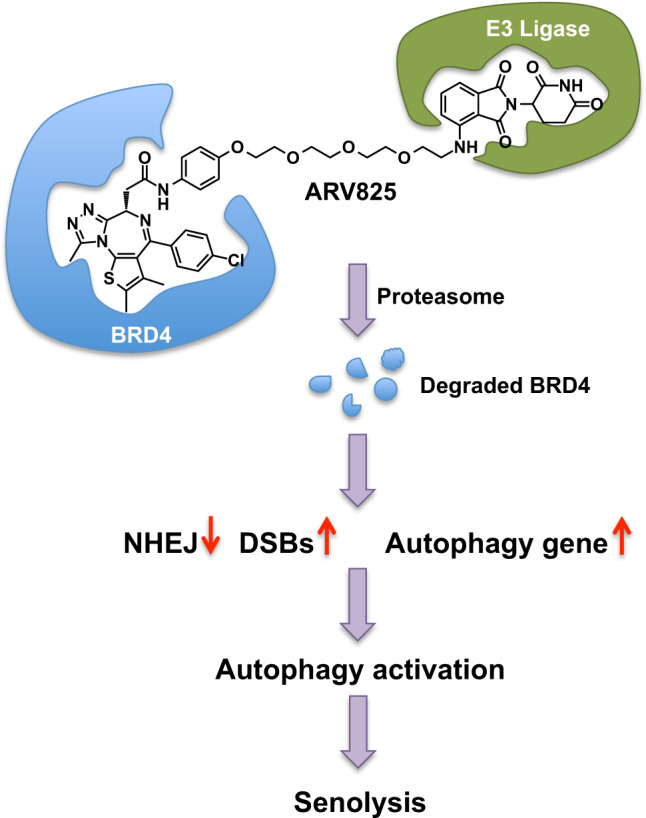
A proposed mechanism for the senolytic activity of BETd ARV825. ARV825 was found to hijack E3 ligase, leading to degradation of BRD4, inhibition of NHEJ and activation of autophagy, and thus induction of senolysis in senescent cells

In a recent study published in *Nature Communications*, Wakita et al. identified BET family protein degrader (BETd) as a novel senolytic drug through high-throughput screening and bio-functional assays.^1^ BETd preferentially eliminated senescent cells by targeting nonhomologus end joining (NHEJ) and autophagy and significantly reduced tumor growth in vivo, suggesting that BETd could be used as a new therapy against cancer and age-related disease (Fig. 1).

Cellular senescence, originally described by Hayfilck and Moorhead,^2^ is a state of stable cell cycle arrest occurring in response to damage and stress and primarily acts as a tumor suppression mechanism. However, the accumulation of senescent cells in multiple organs with age have detrimental effects and eventually contribute to many age-related diseases including cancer, largely due to the development of senescence-associated secretory phenotype (SASP).^3^ The elimination of senescent cells has been shown to not only prevent or treat age-related diseases but also decrease the toxicity of chemotherapy.^4^ Extensive efforts have been made to discover small molecules that can selectively eliminate senescent cells, namely senolytics, as leads for developing novel drugs for age-related diseases.^3^ Although a number of senolytic drugs have been reported, many of these compounds suffer from excess toxicity or cell-type specificity, highlighting the need for the discovery of novel senolytic drugs.

To identify more effective senolytic drugs, Wakita et al. first performed an unbiased high-throughput screening of a chemical library consisting of 47,000 small molecules on Ras-induced senescent cells using a cell viability assay. A total of 15 small molecules were found to selectively induce cell death in senescent cells. Among those hits, four compounds including JQ1 belong to inhibitors of BET family proteins. Wakita et al. next compared the senolytic activities of JQ1, OTX015, a more potent analog of JQ1, and ARV825, a recently developed BETd comprising OTX015 and E3 ligase binder pomalidomide,^5^ to several previously reported senolytic drugs. ARV825 showed the strongest senolytic activity and killed senescent cells at a single-digit nanomolar concentration, regardless of the manner of cellular senescence induction.

To determine whether ARV825 is efficacious in vivo, Wakita et al. first employed an obesity-induced hepatocellular carcinoma (HCC) mouse model. In this model, the increased level of a gut bacterial metabolite deoxycholic acid in mice was capable of causing DNA damage, provoking cellular senescence, and SASP in hepatic stellate cells. This in turn promotes HCC development in neighboring hepatocytes. Compared with the vehicle control group, the ARV825 treatment significantly reduced HCC development as well as the senescent HSC number. Wakita et al. next assessed the senolytic activity of ARV825 in chemotherapy-induced senescent cells. Interestingly, the ARV825 treatment preferentially killed doxorubicin (DXR)-induced senescent cells. Furthermore, compared with DXR treatment alone, combination treatment of ARV825 and DXR significantly reduced the tumor size in mice. Together, these results suggested that ARV825 is efficacious in vivo and capable of not only blocking tumor growth but also increasing the efficacy of chemotherapy.

Having demonstrated the efficacy of ARV825 in vivo, Wakita et al. proceeded to investigate the underlying mechanisms of ARV825’s senolytic action. ARV825 is a hetero-bifunctional Proteolysis Targeting Chimera that hijacks the E3 ubiquitin ligase to degrade BET family proteins.^5^ Wakita et al. first identified the long isoform of BRD4 as the major senolysis target of ARV825 and ARV825 treatment downregulated the *xrcc4* gene, which encodes a protein involved in NHEJ repair for DNA double-strand breaks (DSBs). Moreover, the ARV825 treatment was found to cause the elevation of DSBs and blocked the recruitment of 53BP1 to DSBs in senescent cells. 53BP1 is a DNA damage response protein and the recruitment of 53BP1 to DSBs plays an important role in NHEJ machinery. Therefore, Wakita et al. proposed for the first time that ARV825 may induce senolysis through targeting two independent mechanisms of the NHEJ machinery in senescent cells: inhibiting the *xrcc4* gene expression and blocking the recruitment of 53BP1 to DSB sites.

BRD4 inhibition has been reported to induce autophagic gene expression. Wakita et al. next tested if the activation of autophagy is required for ARV-induced senolysis. Interestingly, the ARV825 treatment was found to increase the expression of a series of autophagic genes and the number of LC3 puncta, a sign of autophagy activation. In addition, ARV825-induced senolysis was significantly reduced by autophagy inhibitor treatment. Moreover, the ARV825 treatment was capable of increasing cleaved caspase-3, an apoptosis marker, as well as LC3B, an autophagy marker, in tumor tissues. Furthermore, co-treatment of autophagy inhibitor and ARV825 significantly attenuated the tumor suppressive effect of ARV825 in mice. In addition to attenuating ARV825-induced senolysis, autophagy inhibitor treatment decreased the activated caspase-3 levels. Lastly, the caspase inhibitor treatment decreased ARV825-induced senolysis. Taken together, these results strongly suggested that the activation of the autophagy is required and autophagy machinery preceded the apoptosis in ARV825-induced senolysis.

In summary, the study by Wakita et al. disclosed ARV825 as a novel and potent senolytic drug. More importantly, the authors elegantly demonstrated that ARV825 is efficacious in vivo and exhibits senolytic activity through targeting NHEJ and autophagy. This study sheds light on the vulnerability of senescent cells and opens up possibilities for its control. However, it also raises several new questions and avenues for future research. For example, does SASP factor contribute to the ARV825-induced senolysis? Is the senolytic effect of ARV825 common to all types of senescent cells in vivo? Is it possible to take advantage of tissue- or cell-specific expression of E3 ligases to achieve the specific cancer treatment in vivo? Does ARV825-induced senolysis have beneficial effects on other age-related diseases? Future studies should pursue answers to these questions and provide a deeper understanding of ARV825-induced senolysis. Overall, the study by Wakita et al. is well designed to yield promising pre-clinical results and solid evidence supporting its novel mechanism. It will be interesting to translate these findings into the clinic.
